# The ability of an oral formulation of afoxolaner to block the transmission of *Babesia canis* by *Dermacentor reticulatus* ticks to dogs

**DOI:** 10.1186/1756-3305-7-283

**Published:** 2014-06-23

**Authors:** Frederic Beugnet, Lenaig Halos, Diane Larsen, Michel Labuschagné, Heidi Erasmus, Josephus Fourie

**Affiliations:** 1 Merial S.A.S., 29 Av Tony Garnier, 69007 Lyon, France; 2 ClinVet International (Pty) Ltd, P.O. Box 11186, Universitas, Bloemfontein 9321, Republic of South Africa

**Keywords:** *Babesia canis*, Dogs, *Dermacentor reticulatus*, Transmission blocking, Afoxolaner

## Abstract

**Background:**

Canine babesiosis due to *Babesia canis* is an endemic disease in many European countries. A vaccine is available in some countries, but it does not prevent the infection and just helps in reducing the gravity of clinical signs. Therefore, the major way to help preventing the disease is by controlling tick infestations on dogs.

To assess the preventive efficacy of afoxolaner (NexGard®), a new oral anti- flea and tick product, against *Babesia canis* infected adult *Dermacentor reticulatus* in an experimentally controlled study.

**Methods:**

Sixteen healthy mixed breed adult dogs, negative for *Babesia canis* antibodies were included in a single centre, randomized, blinded and controlled study to evaluate the impact of treatment with afoxolaner on the transmission of *Babesia canis* to dogs exposed to *Dermacentor reticulatus*. The dogs were randomly allocated into two groups of 8 dogs each. One group remained untreated. In the other group, dogs were treated orally with a novel formulation of afoxolaner (NexGard®) on day 0. All dogs were infested each by 50 adult *Dermacentor reticulatus* ticks (equal sex ratio) at days 7, 14, 21 and 28. The *Dermacentor reticulatus* ticks were confirmed to harbour *Babesia canis* by Polymerase Chain Reaction (PCR).

**Results:**

The treatment was well tolerated by all dogs without any adverse effects. *Babesia canis* was transmitted by *D. reticulatus* to all untreated control dogs, confirmed following demonstration of hyperthermia, detection of *B. canis* parasites in blood smears and PCR assay from blood and serology. These confirmed infected dogs were subsequently treated with imidocarb and diminazene. The treated dogs remained negative based on all criteria until the last study, Day 56, confirming that the oral treatment of dogs with NexGard® prevented transmission of *Babesia canis* and development of clinical babesiosis for up to 28 days.

**Conclusion:**

This is the first demonstration that an oral acaricidal treatment may prevent the transmission of a pathogen despite the need for the tick to attach and start feeding before being killed by the acaricide.

## Background

Ticks are endemic throughout Europe with more than twelve different species, of varying biology and geographic distribution [1]. *Ixodes ricinus* and *Dermacentor reticulatus* commonly infest dogs and are vectors of various canine vector borne pathogens including *Borrelia burgdorferi* and *Anaplasma phagocytophilum,* both transmitted by *I. ricinus,* and *Babesia canis* transmitted by *D. reticulatus*[1,2]. *D. reticulatus* is widely distributed with areas of tick concentration dependent upon local environmental conditions [3]. Its distribution has been expanding in Europe [3-6]. The period of tick activity is also increasing in Europe. For example, the total duration of *Dermacentor* questing activity over the year as well as its presence in winter was shown to increase in Belgium, Switzerland, Poland, Germany, Slovenia and Slovakia [1,7-9]. Climate change is one of the factors which could explain the change in distribution and in activity amongst many others such as land use and host distribution [8,9]. Canine babesiosis is a clinically significant tick-borne protozoan disease, which occurs worldwide. Historically *Babesia* parasites in dogs were divided into two morphologically distinct groups, the larger *Babesia canis* and the smaller *Babesia gibsoni. B. canis* has been reclassified into three sub-species (*B. canis canis, B. canis rossi and B. canis vogeli*) on the basis of vector-specificity and cross-immunity and are now considered to be separate species, *B. canis, B. rossi* and *B. vogeli*[10-12]. However, both species and sub-species names remain in use in the current literature. *Babesia canis* is widely distributed throughout Europe, where it is transmitted by adult *Dermacentor reticulatus* ticks [13-17]. France is the most endemic country in Europe [18,19]. The clinical signs of babesiosis in dogs vary from a mild transient illness to acute disease due to severe haemolysis that rapidly results in death. Clinical findings include anorexia, pale mucous membranes, icterus, pyrexia, and splenic enlargement.

Published studies demonstrating the utility of tick control compounds on dogs have been focused on their acaricidal efficacy against a broad range of ixodid tick species [20]. Some experimental studies have shown the protective effect provided by topical insecticide/acaricide. Efficacy is based on repellent/irritant effect by contact, inhibition of attachment and blood meal, and/or a quick speed of kill [21]. The protective effect of an acaricidal molecule given orally to dogs and acting systemically is less obvious as the ticks must attach and start to feed before being killed. Nevertheless, it has been demonstrated that pathogens need some time to be transmitted [1]. Nevertheless, an acaricide may cause rapid death after ingestion and or block feeding physiology of the tick. The present study describes the results of an experimental study to assess the efficacy of afoxolaner*,* a new insecticide-acaricide administered orally in a soft chewable formulation (Nexgard®, Merial), against *D. reticulatus* infected with *Babesia canis* in dogs. Afoxolaner belongs to a new class of insecticides-acaricides, the isoxazoline [22], acting systemically through oral formulation [23]. Afoxolaner is highly bound to plasmatic proteins and it is ingested by the hematophagous arthropods during their blood meal. It acts as a ligand to a specific receptor on both GABA and glutamate receptors on the ion chloride channel in the neuron synapses. It induces hyperexcitation before death of both fleas and ticks. The new oral formulation, NexGard®, has been proven to control flea and tick infestation (*Ixodes*, *Dermacentor* and *Rhipicephalus*) for a month on dogs [24].

## Methods

### Study design

The study was conducted according to the International Cooperation on Harmonization of Technical Requirements for Registration of Veterinary Medicinal Products Guideline 9: Good Clinical Practice, and in compliance with South African animal welfare legislation. The study employed a controlled, blinded, randomized block design and utilized adult, healthy, mongrel dogs. All dogs were individually penned in tick-proof kennels, were managed similarly and observed once daily for health abnormalities throughout the study. When health abnormalities were detected between the scheduled physical examinations, additional examinations were conducted. In order to control bias, the animals were not treated by a person involved in performing the post-administration assessments and observations.

Two groups of 8 dogs were included, one untreated control and one NexGard® (Merial) treated group. The treatment was administered on Day 0. All dogs were infested by ticks on Days 7, 14, 21 and 28. The ticks were not removed and counted until Day 30. The dogs were blood sampled on Days −7, 14, 21, 28, 35, 42, 49, 56. At Day 56, the last study day, dogs were removed from their boxes and put back to their original runs. In order to check the maintenance of seropositivity in the infected dogs, a blood sample was taken for serology on Day 93.

### Animals

On Day −1, body weight of each dog was used for ranking and group allocation purposes. The study followed a randomised block design. The 16 dogs included on Day −7 were ranked, within sex, in descending order of individual body weight. Within each block, dogs were randomly allocated to groups 1 or 2. The dogs were clinically healthy as verified by a veterinarian on Day −7; they were older than 2 months; they weighed 11.97 kg to 21.43 kg; the females were not pregnant; and they had not been treated with a long acting topical or systemic acaricide/insecticide during the 12 weeks preceding Day −7. All the animals were observed daily from Days −7 to 56.

They were tested PCR negative and sero-negative (IFAT) for *Babesia canis* on Day −7 at the start of acclimatization. They were then maintained individually in cages in a controlled tick free zone where all experimental tick studies are conducted, in order to avoid any external contamination.

During the study, the dogs were examined clinically daily to detect any signs of canine babesiosis including body temperature > 39.4°C.

In addition to scheduled blood samples, a temperature > 39.4°C was the trigger for collection of a blood sample, an immediate blood smear and a PCR. All dogs confirmed positive for *Babesia* by blood smear received the appropriate treatment with imidocarb (Forray® 65) and diminazene (Berenil® RTU). By experience, the combination of imidocarb and diminazene is chosen to allow a single treatment without repeat treatment 14 days later, and to minimize the risk of relapse. Tick challenges were discontinued for these animals.

Dog cages were part of an indoor animal unit in a tick free kennel, environmentally controlled for temperature (20 ± 4°C). The study animals were kept individually in cages and no direct contact between dogs was possible during the study.

### Treatment

Eight dogs were treated on Day 0 with NexGard®, an oral formulation of 2.27% w/w afoxolaner. They were dosed orally by giving a palatable chew by hand. They all ingested the chew spontaneously when offered. All dogs weighed between 10.1 to 25 kg and received the 3 g chew containing 68 mg afoxolaner. No vomiting was observed and none of the animals was redosed.

### Tick challenges

A laboratory-bred *Dermacentor reticulatus* tick strain infected with *Babesia canis* was used for the challenges. The *Dermacentor reticulatus* strain is maintained on dogs and derived from wild ticks collected in Europe. The *D. reticulatus* ticks were infected with *B. canis* by feeding them on dogs previously infected intravenously by with a *B. canis* strain originated from France. The *Babesia* strain was firstly isolated from a *D. reticulatus* female collected from a dog in France. It is conserved in frozen EDTA dog blood and injected to dogs when needed for maintenance or to initiate studies.

A sample of 50 *D. reticulatus* ticks was taken weekly from the batch of ticks to be used for challenges and the infectivity was tested and confirmed by PCR analysis before the infestation of the dogs (Table 1). All dogs were infested with 50 ticks each on Days 7, 14, 21 and 28. Adult ticks, which were used in the challenges, were unfed, at least one week old and had a balanced sex ratio (50% female: 50% male). For logistic reason, the tick infestations of the second week were split between Day 14 and Day 15.

**Table 1:** **Results of the weekly *Babesia canis *** **PCR on 50 ticks from the batch used for dog infestations**

	**Day 7**	**Day 14**	**Day 21**	**Day 28**
**PCR results**	10%	10%	8%	10%

Ticks were applied directly on the dog by tapping the vial to dislodge the ticks from the container so that they could be placed or spread directly over the dog’s hair coat. Gloved fingers were used to facilitate hair penetration by ticks once placed on the animal. Dogs were placed for approximately four hours in an infestation crate to enhance tick attachment, then they were placed again in their individual box. Ticks dislodged during the first 10 minutes were placed back on the dog.

### Tick removal and count

The ticks were removed and counted on dogs diagnosed for babesiosis and on all remaining dogs on Day 30. They were categorized as free or attached and dead or alive, following WAAVP recommendation [25].

### Blood collection and blood smear for Babesia canis detection

In addition to scheduled blood samples, a blood sample was collected and a blood smear prepared in case of clinical suspicion of *Babesia* infection (body temperature > 39.4°C). Blood sampling was realized on the cephalic vein of the arm. Blood was collected for PCR analysis and serology from all dogs diagnosed with babesiosis (on the day of diagnosis, before rescue treatment) based on blood smears and from all dogs on Days - 7, 14, 21, 28, 35, 42, 49, 56. A Day 93 post-study sample was taken on all dogs to check the maintenance of the serological status.

Three-milliliter blood samples were collected in EDTA tubes for PCR analysis. Prior to starting the procedure, approximately 1 mL of blood was taken from the 3 mL whole blood sample and stored in a cryo tube in a −80°C freezer (<70°C), which served as a secondary sample for PCR analysis. The remaining 2 mL whole blood samples were transferred to the ClinVet molecular laboratory for analysis. Total DNA was isolated from individual ticks (for batch infectivity determination and verification) and 200 μl whole blood (for diagnostic purposes) using a commercially available DNA isolation kit. Up to 400 ng isolated DNA served as template in a PCR assay based on targeting 18 S rRNA ITS-1 gene regions originally identified by Duarte *et al.*[26]. The following primers: *Babesia canis* 2F (5′-GGAAGGAGAAGTCGTAACAAGGTTTCC-3′) and *B.canis* 2R (5′-CAGTGGTCACAGACCGGTCG-3′) were used. They are species specific and there is no cross-reaction with *Babesia vogeli* and *Babesia rossi*. The PCR method was developed and tested prior to the study at ClinVet. *Babesia* species have 2 to 5 copies of the rDNA targeted region present per genome [27]. Each set of species specific primers was tested against two individually confirmed cases each for *B. canis* and *B. vogeli* and one confirmed and one suspected case of *B. rossi*. During this study, each PCR run included a positive control, negative control and a no template control. An internal amplification control was employed to validate each PCR reaction success in order to eliminate false negatives. The presence of an approximately 300 bp PCR product, subsequent to agarose gel electrophoresis, confirmed detection of *Babesia canis* DNA in the sample. The obtained PCR products were processed for sequence analysis. Both strands were completely sequenced and assembled sequence data was subjected to BLAST analysis where a 100% identity was obtained towards known *B. canis* sequence data (GenBank accession number: AF394533) as well as the *Babesia canis* originally used for donor animal infection and then to infect the *D. reticulatus* tick vector.

Another 3 mL of blood was collected from each dog for serology. Serum was recovered from the plain tubes and divided into primary and duplicate aliquots. Primary aliquots were stored at 2°C to 8°C for two days until assayed for *Babesia canis* antibodies, using a commercial IFA test carried out as described by Uilenberg *et al.*[10]. Duplicate aliquots were frozen at < −35°C. For screening purposes the sera were diluted at 1:80 and results were expressed as positive (fluorescence at dilution 1:80) or negative (no fluorescence). One positive IFA result was considered sufficient and therefore testing of serum was discontinued after a first positive result on a dog. Nevertheless a post-study Day 93 serology was performed on a blood sample from each dog.

### Effectiveness assessment

The primary assessment criterion was the number of dogs infected with *Babesia canis* in the control and treated group.

### Statistical analysis

An efficacy failure (successful infection with Babesia) was regarded as a dog in the Nexgard® treated group that was tested serologically positive for Babesia canis antibodies or tested positive for Babesia canis by PCR analysis or tested positive by blood smear direct examination.

The infection rate was calculated in the treated group at the end of the study, Day 56, and was compared statistically with that of the control group.

The percentage blocking efficacy was calculated as follows:

$$\mathrm{Efficacy}\left(\%\right)=100\times \left(\mathrm{Tc}-\mathrm{Tt}\right)/\mathrm{Tc},$$

where: Tc = Total number of infected dogs in the negative control groupTt = Total number of infected dogs in the NexGard® administration group.

The proportion of animals infected in each group was also compared using the chi-square test or Fisher’s exact test as applicable. SAS® Version 9.3 TS Level 1 M2 was used for all the statistical analyses. The level of significance of the formal tests was set at 5%, all tests were two sided.

## Results

### Tick infectivity

A sample of 50 *D. reticulatus* ticks was taken from the batch of ticks to be used for each artificial challenge and the infectivity confirmed by PCR analysis. Results of the PCR analysis indicated that 8 – 10% of ticks were *Babesia* infected (Table 1). The arithmetic mean tick counts recorded for the untreated control group 1 was 15.0 at Day 9 and 41.1 on Day 16, indicating a vigorous tick challenge.

### Blood smear evaluation results

In addition to scheduled blood smears at the start of the study, blood smears were prepared and examined for the presence of *B. canis* sporozoites for all dogs with pyrexia (>39.4°C) from Day 7 onwards after first tick infestation (Table 2). *Babesia canis* sporozoites were observed on blood smears from all dogs in the untreated control group on at least one occasion. No parasites were observed on any of the blood smears from the treated dogs.

**Table 2:** Results of blood smear examination of dogs having a temperature > 39.4°C on a daily observation

**Group**	**Body temp range (°C)**		**Blood smear preparation and examination day**										
	**Min**	**Max**	**14**	**15**	**20**	**21**	**25**	**26**	**28**	**29**	**35**	**42**	**49**
1	38.0	40.8	NEG	**POS**	-	-	-	-	-	-	-	-	-
	37.7	39.7	-	**POS**	-	-	-	-	NEG	-	NEG	-	-
	38.0	39.6	**POS**	NEG	-	-	-	-	-	-	NEG	NEG	-
	38.1	39.8	**POS**	NEG	-	-	-	-	-	-	-	NEG	-
	38.2	39.8	-	**POS**	-	-	-	-	-	-	-	-	-
	37.0	40.4	NEG	**POS**	-	-	-	-	-	-	-	-	-
	38.2	39.5	-	**POS**	-	-	-	-	-	-	-	NEG	-
	37.5	40.4	NEG	**POS**	-	-	-	-	-	-	-	-	-
2	37.6	39.5	-	-	-	NEG	NEG	-	-	-	-	NEG	-
	37.5	39.2	-	-	-	-	-	-	-	-	-	NEG	-
	37.9	40.0	NEG	NEG	NEG	-	-	-	NEG	-	-	NEG	NEG
	38.0	39.3	-	-	-	-	-	-	-	-	-	-	-
	37.8	41.3	NEG	NEG	NEG	NEG	-	-	NEG	-	NEG	NEG	NEG
	38.1	39.4	-	-	-	-	-	-	NEG	-	NEG	NEG	-
	37.9	39.4	-	-	-	-	-	-	NEG	-	-	-	-
	37.6	40.1	NEG	NEG	NEG	-	-	NEG	NEG	NEG	NEG	NEG	NEG

Positive results are in bold.

### Tick counts and tick count efficacy data

Live ticks were present on all control dogs at the time they were diagnosed positive for *Babesia* (Day 15 – 16) (Table 2). Infestations were relatively high (8 – 67 ticks). In contrast, only a few dead ticks (0 – 6) were observed on treated dogs on Day 30 at the end of tick phase of the study. This reinforces the efficacy of afoxolaner against *Dermacentor reticulatus* ticks (Table 3). The count and the categorization of ticks on the treated dogs showed that only a few dead attached ticks were observed.

**Table 3:** Results of tick counts on dogs

Study day*	**Group**	**Live: male free**	**Live: female free**	**Live: male attached**	**Live: female attached**	**Dead: male free**	**Dead: female free**	**Dead: male attached**	**Dead: female attached**
15	Control	0	0	**32**	**35**	0	0	0	0
15	Control	0	0	**28**	**31**	0	0	0	0
16	Control	**1**	0	**16**	**15**	**2**	0	0	0
16	Control	0	0	**25**	**24**	**1**	0	**1**	0
16	Control	0	0	**17**	**18**	0	0	0	0
16	Control	0	0	**24**	**24**	0	0	2	0
16	Control	0	0	**6**	**10**	2	0	**1**	**1**
16	Control	0	0	**16**	**16**	0	**1**	0	0
**30**	Treated	0	0	0	0	**2**	**3**	0	0
**30**	Treated	0	0	0	0	**1**	**1**	0	0
**30**	Treated	0	0	0	0	**2**	**2**	0	**1**
**30**	Treated	0	0	0	0	0	**1**	0	0
**30**	Treated	0	0	0	0	0	0	**2**	0
**30**	Treated	0	0	0	0	**3**	**2**	**1**	0
**30**	Treated	0	0	0	0	0	0	**1**	0
**30**	Treated	0	0	0	0	0	0	0	0

*Study day where the dogs were removed due to canine babesiosis; or Day 30, 48 h after the last tick infestation.

Tick numbers are indicated in bold when it is >0.

### Babesia canis infection

All dogs in the untreated control group were positive for babesiosis based on blood smear and PCR analysis. Seven out of the eight control dogs became serologically positive on Day 21 and remained positive until the last study Day 56 (Table 4). All control dogs were *Babesia* infected after the two tick infestations on Day 7 or Day 14/15. None of the dogs in the treated group was positive for babesiosis on blood smear, IFAT or PCR analysis during the 56 days of the study. Nexgard® treatment was therefore regarded to be fully effective in preventing the transmission of *Babesia canis* by infected *Dermacentor reticulatus* ticks (100% preventive efficacy, p = 0.0014).

**Table 4:** **Summary of assessment for *B. canis *** **infection**

**Group**	**- 7**	**14/15**		**+21**		**+28**		**+35**		**+42**		**+49**		**+56 (last study Day)**		**+ 93**
	**IFAT**	**IFAT**	**PCR**	**IFAT**	**PCR**	**IFAT**	**PCR**	**IFAT**	**PCR**	**IFAT**	**PCR**	**IFAT**	**PCR**	**IFAT**	**PCR**	**IFAT**
Control	NEG	NEG	**POS**	**POS**	**POS**		**POS**		NEG		NEG		NEG		NEG	**POS**
	NEG	NEG	**POS**	**POS**	NEG		NEG		NEG		NEG		NEG		NEG	**POS**
	NEG	NEG	**POS**	**POS**	NEG		NEG		NEG		NEG		NEG		NEG	**POS**
	NEG	NEG	**POS**	**POS**	NEG		NEG		NEG		NEG		NEG		NEG	**POS**
	NEG	NEG	**POS**	**POS**	NEG		NEG		NEG		NEG		NEG		NEG	**POS**
	NEG	NEG	**POS**	**POS**	**POS**		NEG		NEG		NEG		NEG		NEG	**POS**
	NEG	NEG	**POS**	NEG	NEG	NEG	NEG	NEG	NEG	NEG	NEG	NEG	NEG	NEG	NEG	**NEG**
	NEG	NEG	**POS**	**POS**	NEG		NEG		NEG		NEG		NEG		NEG	**POS**
Treated	NEG	NEG	NEG	NEG	NEG	NEG	NEG	NEG	NEG	NEG	NEG	NEG	NEG	**NEG**	**NEG**	+/− POS*
	NEG	NEG	NEG	NEG	NEG	NEG	NEG	NEG	NEG	NEG	NEG	NEG	NEG	**NEG**	**NEG**	**NEG**
	NEG	NEG	NEG	NEG	NEG	NEG	NEG	NEG	NEG	NEG	NEG	NEG	NEG	**NEG**	**NEG**	**NEG**
	NEG	NEG	NEG	NEG	NEG	NEG	NEG	NEG	NEG	NEG	NEG	NEG	NEG	**NEG**	**NEG**	**NEG**
	NEG	NEG	NEG	NEG	NEG	NEG	NEG	NEG	NEG	NEG	NEG	NEG	NEG	**NEG**	**NEG**	**NEG**
	NEG	NEG	NEG	NEG	NEG	NEG	NEG	NEG	NEG	NEG	NEG	NEG	NEG	**NEG**	**NEG**	**NEG**
	NEG	NEG	NEG	NEG	NEG	NEG	NEG	NEG	NEG	NEG	NEG	NEG	NEG	**NEG**	**NEG**	**NEG**
	NEG	NEG	NEG	NEG	NEG	NEG	NEG	NEG	NEG	NEG	NEG	NEG	NEG	**NEG**	**NEG**	**NEG**

*This dog did not show any clinical signs, and remained Blood smear, PCR and serology negative until the end of the study (Day 56). He was found to be positive by one examiner at Day 93, showing a diffuse fluorescence including dog cells and some *Babesia canis*, which was considered as negative by another examiner.

Positive results and final check at Day 56 and Day 93 are indicated in bold.

At Day 93, the 7 seropositive control dogs were still positive. Surprisingly, one previously negative treated dog was found slightly positive by IFAT (considered positive by one examiner and negative by another). PCR and blood smear were negative. This particular dog was negative for blood smear, PCR and serology throughout the study until day 56 and did not show any clinical signs of infection.

## Discussion

Conduct of this study required the generation of a large batch of ticks with an adequate *Babesia* infection rate. The rates of infection calculated by PCR on ticks were 8-10% during one month. This is similar to what was described by Fourie *et al*. [28] assessing the efficacy of the combination of fipronil-amitraz-(S)-methoprene (Certifect®). Regardless of which sex or if both sexes transmit the *B. canis,* this infection rate in the ticks was sufficiently high to successfully transmit the infection to 8 out of 8 untreated (control) dogs. All untreated dogs were infected and were rescue treated immediately after diagnosis. The early treatment can explain the absence of the classical clinical signs of babesiosis, including fever syndrome, anemia, hemolysis and hemoglobinuria. It also possibly explains that one control dog, despite a positive blood smear and PCR remained serologically negative. Regarding the treated dog that possibly seroconverted on Day 93 post-study, as all dogs were moved back to their community runs on Day 56, it is not known whether it is a late seroconversion, a more recent infection that appeared after the study, or a false positive fluorescence due to another reason.

Protozoan parasites require additional time, usually several days, for their sporoblasts to mature into sporozoites in the salivary glands of the tick before they can be secreted into the saliva and transmitted to the mammalian host. For instance, *Babesia microti* is transmitted by *Ixodes scapularis* between 36 and 48 hours after tick attachment [29]. The treatment assessed here is an oral formulation, meaning that ticks need to attach and start to feed before being killed. Afoxolaner efficacy against *Dermacentor reticulatus* has been demonstrated for a full month [24] with ticks evaluated at 48 hours after each infestation. The protection against the transmission of *Babesia canis* demonstrated in this study is a consequence of the time needed for ticks to transmit *Babesia*. It may also be due to the action of afoxolaner on the tick feeding physiology or on the rapid tick death. The fact that no live ticks were found at Day 28 and very few attached ticks is suggestive of a quick death but also detachment, which may also impact on the transmission of *Babesia*. Another study involving the combination of Fipronil-amitraz-(S)-methoprene demonstrated 86% efficacy against transmission using a similar protocol [21]. This topical formulation is known to induce a quick death, in less than 24 h and a detachment or a prevention of attachment of ticks [30].

## Conclusion

The oral formulation of afoxolaner demonstrated in this experimental study a complete efficacy in preventing the transmission of *Babesia canis*.

## Competing interest

The work reported herein was funded by Merial SAS, France. The authors are current employees or contractors of Merial.

®CERTIFECT and NEXGARD are registered trademarks of Merial. All other marks are the property of their respective owners.

This document is provided for scientific purposes only. Any reference to a brand or trademark herein is for informational purposes only and is not intended for a commercial purpose or to dilute the rights of the respective owner(s) of the brand(s) and trademark(s).

## Authors’ contributions

All authors read and approved the final version of the manuscript.
